# Ethyl 3-(10-bromo­anthracen-9-yl)-5-methyl-1,2-oxazole-4-carboxyl­ate

**DOI:** 10.1107/S1600536813031395

**Published:** 2013-11-23

**Authors:** Chun Li, Michael J. Campbell, Matthew J. Weaver, Nathan S. Duncan, Janet L. Hunting, Nicholas R. Natale

**Affiliations:** a Department of Chemistry, Ithaca College, 953 Danby Road, Ithaca, NY 14850, USA; b Department of Pharmaceutical & Biomedical Science, The University of Montana, 32 Campus Drive, Missoula, MT 59812, USA

## Abstract

In the title compound, C_21_H_16_BrNO_3_, the mean planes of the anthracene tricycle and isoxazole ring are inclined to each other at a dihedral angle of 72.12 (7)°. The carb­oxy group is slightly out of the isoxazole mean plane, with a maximum deviation of 0.070 (5) Å for the carbonyl O atom. In the crystal, pairs of weak C—H⋯O hydrogen bonds link the mol­ecules into dimers, and weak C—H⋯N inter­actions further link these dimers into corrugated layers parallel to the *bc* plane.

## Related literature

For the synthesis of anthryl isoxazoles, see: Mosher & Natale (1995 ▶); Zhou *et al.* (1997 ▶); Han & Natale (2001 ▶); Rider *et al.* (2010 ▶); Mirzaei *et al.* (2012 ▶). For related structures, see: Mosher *et al.* (1996 ▶); Han *et al.* (2002 ▶, 2003 ▶); Li *et al.* (2006 ▶, 2008 ▶). For the anti­tumor activity of aryl isoxazole amides (AIMs), see: Han *et al.* (2009 ▶); Gajewski *et al.* (2009 ▶); Balasubramanian *et al.* (2011 ▶); Neidle (2012 ▶); Kohn *et al.* (2012 ▶); Shoemaker (2006 ▶).
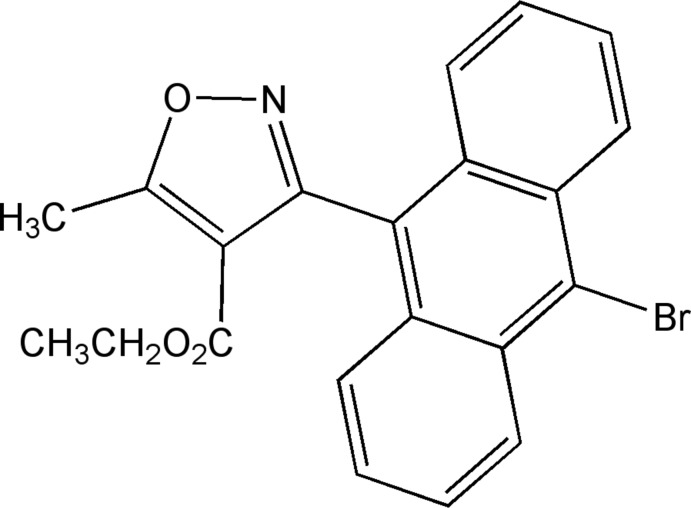


## Experimental

### 

#### Crystal data

C_21_H_16_BrNO_3_
*M* *_r_* = 410.26
Monoclinic, 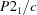

*a* = 8.8437 (1) Å
*b* = 16.7099 (2) Å
*c* = 11.7157 (2) Å
β = 92.419 (1)°
*V* = 1729.77 (4) Å^3^
*Z* = 4
Mo *K*α radiation
μ = 2.40 mm^−1^
*T* = 100 K
0.49 × 0.47 × 0.38 mm

#### Data collection

Bruker SMART BREEZE CCD diffractometer
Absorption correction: multi-scan (*SADABS*; Bruker, 2008 ▶) *T*_min_ = 0.39, *T*_max_ = 0.47
34958 measured reflections
4290 independent reflections
4128 reflections with *I* > 2σ(*I*)
*R*_int_ = 0.020

#### Refinement

*R*[*F* ^2^ > 2σ(*F* ^2^)] = 0.025
*wR*(*F* ^2^) = 0.074
*S* = 1.05
4290 reflections
237 parameters
H-atom parameters constrained
Δρ_max_ = 0.51 e Å^−3^
Δρ_min_ = −0.39 e Å^−3^

### 

Data collection: *APEX2* (Bruker, 2008 ▶); cell refinement: *SAINT* (Bruker, 2008 ▶); data reduction: *SAINT*; program(s) used to solve structure: *SHELXL97* (Sheldrick, 2008 ▶); program(s) used to refine structure: *SHELXS97* (Sheldrick, 2008 ▶); molecular graphics: *OLEX2* (Dolomanov *et al.*, 2009 ▶); software used to prepare material for publication: *OLEX2* and *publCIF* (Westrip, 2010 ▶).

## Supplementary Material

Crystal structure: contains datablock(s) I. DOI: 10.1107/S1600536813031395/cv5433sup1.cif

Structure factors: contains datablock(s) I. DOI: 10.1107/S1600536813031395/cv5433Isup2.hkl

Supplementary material file. DOI: 10.1107/S1600536813031395/cv5433Isup3.cml

Additional supplementary materials:  crystallographic information; 3D view; checkCIF report

## Figures and Tables

**Table 1:** Hydrogen-bond geometry (Å, °)

*D*—H⋯*A*	*D*—H	H⋯*A*	*D*⋯*A*	*D*—H⋯*A*
C2—H2⋯N1^i^	0.95	2.54	3.4305 (19)	155
C4—H4⋯O2^ii^	0.95	2.58	3.1990 (18)	123

Symmetry codes: (i) 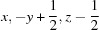; (ii) 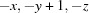.

**Fig. 1.**
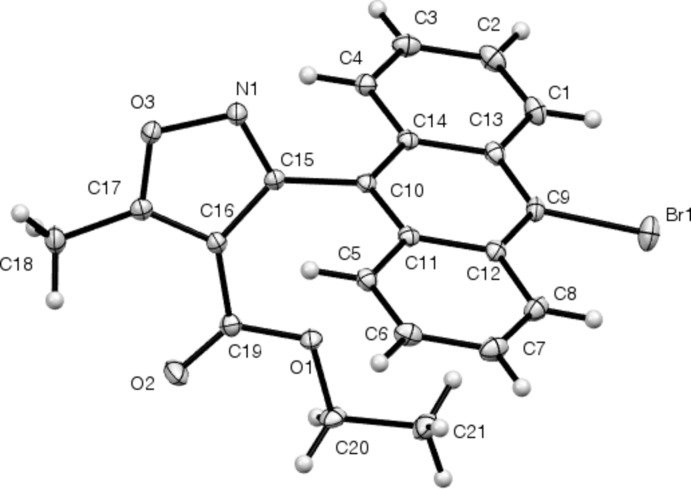
The molecular structure of titled compound, with atom labels and 50% probability displacement ellipsoids for non-H atoms.

**Fig. 2.**
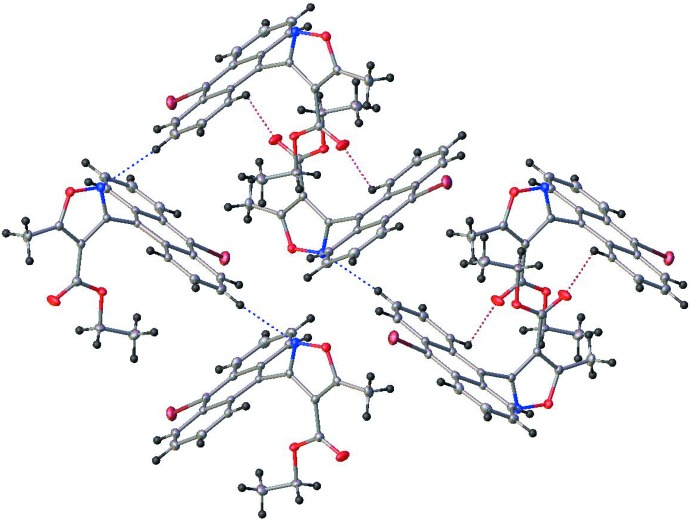
A portion of the packing diagram viewed in [100]. Weak intermolecular C—H···O and C—H···N hydrogen bond interactions are shown as dashed lines.

## supplementary crystallographic information

### 1. Introduction

The aryl isoxazole amides (AIMs) (Han *et al.*, 2009; Gajewski *et al.*, 2009) have significant activity in the National Cancer Institutes 60 cell line screen (Kohn *et al.*, 2012; Shoemaker *et al.*, 2006) comparable to several agents in general medical practice, such as fluoro­uridine and bleomycin. Our working hypothesis for developing the structure activity relationship (SAR) of AIMs to improve their anti-tumor efficacy is focused on the quadruplex DNA conformations (Balasubramanian *et al.*, 2011; Neidle, 2012). To more accurately inform our G-4 - small molecule docking studies, we rely on crystallographic determinations of AIMs.

### 2.1. Synthesis and crystallization

To a suspension of anthracene-9-carbaldehyde (10.0 g, 48.49 mmol; Sigma-Aldrich, 97%) in THF:Ethanol:H2O (135 mL:67.5 mL:67.5 mL) was dissolved sodium acetate (3.5 eq., 13.92 g, 169.7 mmol) and hydroxyl­amine hydro­chloride (2 eq, 6.7387 g, 96.974 mmol). The reaction was covered and let stir at room temperature until TLC showed no starting material remained (ca. 96 hours). The solution was then transferred to a separatory funnel and washed 4 x 350 mL Brine and the combined aqueous layers washed 2x100 mL CHCl_3_, dried over sodium sulfate, filtered, and the solvent removed under vacuum to yield anthracene-9-carbaldehyde oxime (99%). ^1^H NMR(CDCl_3_) δ 9.22 (s, 1H), 8.51 (s, 1H), 8.42 (d, J=8.66 Hz, 2H), 8.03 (d, J=8.16 Hz, 2H), 7.55 (m, 4H).

The anthracene-9-carbaldehyde oxime (10.516 g, 47.53 mmol) was taken up in 200 mL of chloro­form at room temperature, to which solution was added pyridine (10 mol%, 0.38 mL) and recrystallized NCS (1.1 eq., 7.197 g, 52.28 mmol). The solution brought to 40°C for three hours then cooled to room temperature. The organic layer was washed with 4x450 mL Brine and 4x300 mL H_2_O, then the aqueous layer washed with 2x300 mL CHCl_3_, dried with sodium sulfate, filtered, and the solvent removed under reduced pressure to yield the nitrile oxide. The inter­mediate was purified only through extractive isolation using brine and CHCl_3_ and taken on to the next reaction as is. To a solution of the nitrile oxide in absolute ethanol (230 mL) was added 1.4 equivalents of ethyl­aceto­acetate. In a separate flask was added 115 mL absolute ethanol and 2.341 g Na(s). Once the sodium dissociation had completed, the warm solution was added to the nitrile oxide and the mixture was allowed to stir at room temperature under argon for 20 hours until TLC in 4:1 Hex/EtOAc revealed all nitrile oxide had been consumed. Finally, the ethanol was removed via rotary evaporation and the solid chromatographed using 4:1 Hex/EtOAc (R_f_=0.56). Ethyl 3-(anthracen-9-yl)-5-methyl­isoxazole-4-carboxyl­ate. Yield 97%. ^1^H NMR(400 MHz, CDCl_3_) δ 8.59 (s, 1H), 8.06 (d, J=7.91 Hz, 2H), 7.66 (d, J=8.16 Hz, 2H), 7.41-7.50 (m, 4H), 3.70 (q, J=7.15, 14.31 Hz, 2H), 2.93 (s, 3H), 0.33 (t, J=7.15 Hz, 3H). Spectral data are in accord with those reported previously (Mirzaei *et al.*, 2012).

Ethyl 3-(anthracen-9-yl)-5-methyl­isoxazole-4-carboxyl­ate (4.88 g, 14.73 mmol) was taken up in 80 mL DMF to which was added a solution of recrystallized N-Bromo­succinamide (NBS) (1.1 eq, 2.884 g, 16.203 mmol) dissolved in 80 mL DMF. The solution was brought to 40°C and let stir for 5 hours where upon the solution was poured into 1200mL ice/water which was allowed to stir for 2 hours, in which the product precipitated out. Product was filtered, dissolved in CH_2_Cl_2_, dried over anhydrous sodium sulfate, filtered and concentrated under vacuum. Yield 88%, ^1^H NMR(CDCl_3_) δ 8.62 (d, J=8.91 Hz, 2H), 7.60-7.67 (m, 4H), 7.61 (m, 2H), 3.72 (q, J=7.03, 14.18 Hz, 2H), 2.94 (s, 3H), 0.39 (t, J=7.15, 14.31 Hz, 3H); ^13^C NMR(CDCl_3_) δ 176.28, 161.23, 160.21, 131.31, 130.01, 128.03, 127.04, 126.50, 125.87, 125.18, 123.62, 111.40, 60.14, 13.41, 12.89. Spectral data are in accord with those reported previously (Han *et al.*, 2003), the crystals were obtained by slow evapotation from a methyl­ene chloride and heptane solution.

### 2.2. Refinement

All H atoms were placed at geometrically calculated positions and included in the refinement in the riding model approximation, with C—H lengths of 0.95 (aromatic CH), 0.98 (CH_3_) and 0.99 (CH_2_) Å. Idealized methyl groups were refined as rotating groups. *U*_iso_ of the H atoms was set at 1.5*U*_eq_ of the parent C atom for the methyl group and at 1.2*U*_eq_ for the remaining H atoms.

### 3. Results and discussion

The aryl isoxazole amides (AIMs) (Han *et al.*, 2009; Gajewski *et al.*, 2009) have significant activity in the National Cancer Institutes 60 cell line screen, (Kohn *et al.*, 2012; Shoemaker, 2006) comparable to several agents in general medical practice, such as fluoro­uridine and bleomycin. Our working hypothesis for developing the structure activity relationship (SAR) of AIMs to improve their anti-tumor efficacy is focused on the quadruplex DNA conformations (Balasubramanian *et al.*, 2011; Neidle *et al.*, 2012). To more accurately inform our G-4 - small molecule docking studies, we rely on crystallographic determinations of AIMs. In previous studies we have determined that the dihedral angle between the isoxazole and the C-3 aryl is approximately orthogonal (Mosher *et al.*, 1996; Li *et al.*, 2008), and we have postulated that this is a critical feature in the biological activity of the AIMs. The anthracenyl group is almost perpendicular to the isoxazole plane in ethyl 3-(10'-chloro­anthracenyl)-5-(1''-phenyl-2''-hydroxyl­ethyl­enyl)isoxazole-4-carboxyl­ate [85.51 (4)°] (Li *et al.*, 2006), which is similar to analogous anthracenyl isoxazole structures in the Cambridge Structural Database, i.e. ethyl 3-(10'-chloro-9'-anthracenyl)-5-methyl-4-isoxazolcarboxyl­ate [74.3°] (Han *et al.*, 2003; CSD refcode EZENEC), ethyl 3-(10'-chloro-9'-anthracenyl)-5-(2-phenyl­ethyl)-4-isoxazole­carboxyl­ate [78.5°] (Han *et al.*, 2002; CSD refcode MUQMOA). This AIM analog represents a key inter­mediate in future synthesis and SAR studies, and our progress will be reported in due course.

### Crystal data

**Table:**

C_21_H_16_BrNO_3_	*F*(000) = 832
*M**_r_* = 410.26	*D*_x_ = 1.575 Mg m^−^^3^
Monoclinic, *P*2_1_/*c*	Mo *K*α radiation, λ = 0.71073 Å
*a* = 8.8437 (1) Å	Cell parameters from 330 reflections
*b* = 16.7099 (2) Å	θ = 0.3–27.5°
*c* = 11.7157 (2) Å	µ = 2.40 mm^−^^1^
β = 92.419 (1)°	*T* = 100 K
*V* = 1729.77 (4) Å^3^	Prism, translucent yellow
*Z* = 4	0.49 × 0.47 × 0.38 mm

### Data collection

**Table:**

Bruker SMART BREEZE CCD diffractometer	4128 reflections with *I* > 2σ(*I*)
Radiation source: 2 kW sealed X-ray tube	*R*_int_ = 0.020
π and ω scans	θ_max_ = 28.3°, θ_min_ = 2.1°
Absorption correction: multi-scan (*SADABS*; Bruker, 2008)	*h* = −11→11
*T*_min_ = 0.39, *T*_max_ = 0.47	*k* = −22→22
34958 measured reflections	*l* = −15→14
4290 independent reflections	

### Refinement

**Table:**

Refinement on *F*^2^	Primary atom site location: structure-invariant direct methods
Least-squares matrix: full	Hydrogen site location: inferred from neighbouring sites
*R*[*F*^2^ > 2σ(*F*^2^)] = 0.025	H-atom parameters constrained
*wR*(*F*^2^) = 0.074	*w* = 1/[σ^2^(*F*_o_^2^) + (0.0448*P*)^2^ + 1.2294*P*] where *P* = (*F*_o_^2^ + 2*F*_c_^2^)/3
*S* = 1.05	(Δ/σ)_max_ = 0.002
4290 reflections	Δρ_max_ = 0.51 e Å^−^^3^
237 parameters	Δρ_min_ = −0.39 e Å^−^^3^
0 restraints	

### Special details

**Table:**

Geometry. All e.s.d.'s (except the e.s.d. in the dihedral angle between two l.s. planes) are estimated using the full covariance matrix. The cell e.s.d.'s are taken into account individually in the estimation of e.s.d.'s in distances, angles and torsion angles; correlations between e.s.d.'s in cell parameters are only used when they are defined by crystal symmetry. An approximate (isotropic) treatment of cell e.s.d.'s is used for estimating e.s.d.'s involving l.s. planes.
Refinement. Refinement of *F*^2^ against ALL reflections. The weighted *R*-factor *wR* and goodness of fit *S* are based on *F*^2^, conventional *R*-factors *R* are based on *F*, with *F* set to zero for negative *F*^2^. The threshold expression of *F*^2^ > σ(*F*^2^) is used only for calculating *R*-factors(gt) *etc*. and is not relevant to the choice of reflections for refinement. *R*-factors based on *F*^2^ are statistically about twice as large as those based on *F*, and *R*-factors based on ALL data will be even larger.

### Fractional atomic coordinates and isotropic or equivalent isotropic displacement parameters (Å^2^)

**Table:**

	*x*	*y*	*z*	*U*_iso_*/*U*_eq_	
Br1	0.65922 (2)	0.19002 (2)	0.12783 (2)	0.02178 (7)	
O1	0.29601 (12)	0.46676 (6)	0.02087 (8)	0.0145 (2)	
O2	0.14962 (15)	0.57548 (8)	−0.00887 (10)	0.0261 (3)	
O3	0.06569 (12)	0.53843 (6)	0.34089 (9)	0.0145 (2)	
N1	0.15853 (14)	0.46963 (7)	0.35626 (10)	0.0142 (2)	
C1	0.31314 (18)	0.19895 (9)	0.06508 (13)	0.0166 (3)	
H1	0.3800	0.1580	0.0424	0.020*	
C2	0.16567 (19)	0.19731 (9)	0.02681 (13)	0.0187 (3)	
H2	0.1310	0.1554	−0.0224	0.022*	
C3	0.06357 (17)	0.25744 (9)	0.05964 (12)	0.0167 (3)	
H3	−0.0390	0.2555	0.0323	0.020*	
C4	0.11134 (17)	0.31807 (8)	0.13019 (12)	0.0135 (3)	
H4	0.0412	0.3577	0.1520	0.016*	
C5	0.52484 (16)	0.45590 (8)	0.35132 (12)	0.0141 (3)	
H5	0.4571	0.4969	0.3725	0.017*	
C6	0.67305 (17)	0.45991 (9)	0.38810 (12)	0.0171 (3)	
H6	0.7075	0.5034	0.4345	0.020*	
C7	0.77617 (17)	0.39918 (10)	0.35718 (13)	0.0191 (3)	
H7	0.8791	0.4022	0.3834	0.023*	
C8	0.72856 (16)	0.33668 (9)	0.29027 (13)	0.0175 (3)	
H8	0.7993	0.2969	0.2701	0.021*	
C9	0.52055 (16)	0.26791 (8)	0.17830 (12)	0.0133 (2)	
C10	0.31823 (15)	0.38718 (8)	0.23963 (11)	0.0111 (2)	
C11	0.46912 (15)	0.39100 (8)	0.28134 (11)	0.0116 (2)	
C12	0.57441 (16)	0.32977 (8)	0.24980 (12)	0.0132 (3)	
C13	0.36915 (16)	0.26146 (8)	0.13894 (11)	0.0126 (2)	
C14	0.26560 (16)	0.32284 (8)	0.17184 (11)	0.0114 (2)	
C15	0.21490 (15)	0.45584 (8)	0.25655 (11)	0.0111 (2)	
C16	0.16273 (15)	0.51365 (8)	0.17353 (11)	0.0118 (2)	
C17	0.06993 (15)	0.56274 (8)	0.23177 (12)	0.0128 (2)	
C18	−0.02091 (17)	0.63431 (9)	0.19866 (13)	0.0184 (3)	
H18A	−0.1080	0.6384	0.2474	0.028*	
H18B	−0.0567	0.6295	0.1186	0.028*	
H18C	0.0419	0.6824	0.2081	0.028*	
C19	0.19946 (16)	0.52302 (8)	0.05278 (12)	0.0139 (3)	
C20	0.34574 (17)	0.47134 (9)	−0.09567 (12)	0.0162 (3)	
H20A	0.4147	0.5174	−0.1044	0.019*	
H20B	0.2578	0.4778	−0.1500	0.019*	
C21	0.42720 (19)	0.39376 (10)	−0.11786 (13)	0.0211 (3)	
H21A	0.4647	0.3944	−0.1954	0.032*	
H21B	0.3571	0.3488	−0.1102	0.032*	
H21C	0.5126	0.3878	−0.0624	0.032*	

### Atomic displacement parameters (Å^2^)

**Table:**

	*U*^11^	*U*^22^	*U*^33^	*U*^12^	*U*^13^	*U*^23^
Br1	0.01713 (9)	0.01729 (9)	0.03139 (10)	0.00372 (5)	0.00673 (6)	−0.00296 (5)
O1	0.0174 (5)	0.0153 (5)	0.0111 (4)	0.0030 (4)	0.0040 (4)	0.0021 (4)
O2	0.0333 (7)	0.0279 (6)	0.0175 (5)	0.0158 (5)	0.0050 (5)	0.0084 (4)
O3	0.0153 (5)	0.0136 (5)	0.0146 (5)	0.0031 (4)	0.0024 (4)	−0.0010 (4)
N1	0.0152 (6)	0.0125 (5)	0.0150 (5)	0.0029 (4)	0.0015 (4)	0.0002 (4)
C1	0.0210 (7)	0.0129 (6)	0.0162 (6)	−0.0005 (5)	0.0036 (5)	−0.0019 (5)
C2	0.0237 (8)	0.0157 (7)	0.0167 (7)	−0.0045 (5)	0.0010 (6)	−0.0026 (5)
C3	0.0169 (7)	0.0167 (6)	0.0162 (6)	−0.0039 (5)	−0.0020 (5)	0.0016 (5)
C4	0.0143 (7)	0.0125 (6)	0.0135 (6)	0.0005 (5)	0.0004 (5)	0.0020 (5)
C5	0.0157 (6)	0.0142 (6)	0.0124 (6)	0.0002 (5)	0.0003 (5)	0.0002 (5)
C6	0.0177 (7)	0.0183 (7)	0.0150 (6)	−0.0038 (5)	−0.0016 (5)	−0.0002 (5)
C7	0.0128 (6)	0.0222 (7)	0.0219 (7)	−0.0011 (5)	−0.0023 (5)	0.0041 (6)
C8	0.0121 (6)	0.0175 (7)	0.0227 (7)	0.0020 (5)	0.0006 (5)	0.0028 (5)
C9	0.0131 (6)	0.0108 (6)	0.0162 (6)	0.0029 (5)	0.0042 (5)	0.0010 (5)
C10	0.0123 (6)	0.0110 (6)	0.0101 (5)	0.0013 (5)	0.0013 (4)	0.0018 (4)
C11	0.0126 (6)	0.0115 (6)	0.0106 (6)	0.0000 (5)	0.0012 (4)	0.0019 (4)
C12	0.0124 (6)	0.0128 (6)	0.0143 (6)	0.0008 (5)	0.0015 (5)	0.0033 (5)
C13	0.0147 (6)	0.0107 (6)	0.0126 (6)	0.0001 (5)	0.0024 (5)	0.0014 (5)
C14	0.0125 (6)	0.0111 (5)	0.0107 (6)	−0.0004 (5)	0.0013 (5)	0.0021 (5)
C15	0.0108 (6)	0.0104 (6)	0.0122 (6)	−0.0010 (5)	−0.0003 (4)	−0.0007 (4)
C16	0.0114 (6)	0.0111 (6)	0.0129 (6)	0.0004 (5)	−0.0005 (4)	0.0004 (5)
C17	0.0121 (6)	0.0120 (6)	0.0144 (6)	−0.0012 (5)	−0.0008 (5)	−0.0009 (5)
C18	0.0179 (7)	0.0135 (6)	0.0235 (7)	0.0043 (5)	−0.0016 (5)	−0.0019 (5)
C19	0.0132 (6)	0.0145 (6)	0.0139 (6)	0.0001 (5)	0.0006 (5)	−0.0001 (5)
C20	0.0207 (7)	0.0175 (7)	0.0106 (6)	−0.0008 (5)	0.0049 (5)	0.0010 (5)
C21	0.0246 (8)	0.0213 (7)	0.0180 (7)	0.0028 (6)	0.0063 (6)	−0.0027 (6)

### Geometric parameters (Å, º)

**Table:**

Br1—C9	1.8996 (13)	C8—H8	0.9500
O1—C19	1.3340 (17)	C8—C12	1.4290 (19)
O1—C20	1.4539 (16)	C9—C12	1.4013 (19)
O2—C19	1.2073 (18)	C9—C13	1.4017 (19)
O3—N1	1.4197 (15)	C10—C11	1.4031 (18)
O3—C17	1.3435 (17)	C10—C14	1.4043 (19)
N1—C15	1.3097 (18)	C10—C15	1.4853 (18)
C1—H1	0.9500	C11—C12	1.4420 (19)
C1—C2	1.361 (2)	C13—C14	1.4384 (19)
C1—C13	1.4314 (19)	C15—C16	1.4332 (18)
C2—H2	0.9500	C16—C17	1.3636 (19)
C2—C3	1.415 (2)	C16—C19	1.4731 (18)
C3—H3	0.9500	C17—C18	1.4832 (19)
C3—C4	1.363 (2)	C18—H18A	0.9800
C4—H4	0.9500	C18—H18B	0.9800
C4—C14	1.4316 (19)	C18—H18C	0.9800
C5—H5	0.9500	C20—H20A	0.9900
C5—C6	1.364 (2)	C20—H20B	0.9900
C5—C11	1.4342 (19)	C20—C21	1.511 (2)
C6—H6	0.9500	C21—H21A	0.9800
C6—C7	1.422 (2)	C21—H21B	0.9800
C7—H7	0.9500	C21—H21C	0.9800
C7—C8	1.362 (2)		
			
C19—O1—C20	116.66 (11)	C9—C12—C11	118.01 (12)
C17—O3—N1	109.05 (10)	C1—C13—C14	118.32 (13)
C15—N1—O3	105.62 (11)	C9—C13—C1	123.80 (13)
C2—C1—H1	119.5	C9—C13—C14	117.85 (12)
C2—C1—C13	121.07 (14)	C4—C14—C13	118.50 (12)
C13—C1—H1	119.5	C10—C14—C4	121.59 (13)
C1—C2—H2	119.7	C10—C14—C13	119.86 (13)
C1—C2—C3	120.64 (13)	N1—C15—C10	120.96 (12)
C3—C2—H2	119.7	N1—C15—C16	111.28 (12)
C2—C3—H3	119.8	C16—C15—C10	127.76 (12)
C4—C3—C2	120.50 (14)	C15—C16—C19	130.21 (12)
C4—C3—H3	119.8	C17—C16—C15	104.43 (12)
C3—C4—H4	119.5	C17—C16—C19	125.33 (12)
C3—C4—C14	120.97 (13)	O3—C17—C16	109.62 (12)
C14—C4—H4	119.5	O3—C17—C18	117.14 (12)
C6—C5—H5	119.3	C16—C17—C18	133.24 (13)
C6—C5—C11	121.34 (13)	C17—C18—H18A	109.5
C11—C5—H5	119.3	C17—C18—H18B	109.5
C5—C6—H6	119.9	C17—C18—H18C	109.5
C5—C6—C7	120.18 (14)	H18A—C18—H18B	109.5
C7—C6—H6	119.9	H18A—C18—H18C	109.5
C6—C7—H7	119.7	H18B—C18—H18C	109.5
C8—C7—C6	120.54 (13)	O1—C19—C16	111.34 (12)
C8—C7—H7	119.7	O2—C19—O1	124.46 (13)
C7—C8—H8	119.3	O2—C19—C16	124.19 (13)
C7—C8—C12	121.36 (14)	O1—C20—H20A	110.5
C12—C8—H8	119.3	O1—C20—H20B	110.5
C12—C9—Br1	118.94 (10)	O1—C20—C21	106.36 (11)
C12—C9—C13	123.32 (12)	H20A—C20—H20B	108.6
C13—C9—Br1	117.72 (10)	C21—C20—H20A	110.5
C11—C10—C14	121.29 (12)	C21—C20—H20B	110.5
C11—C10—C15	120.00 (12)	C20—C21—H21A	109.5
C14—C10—C15	118.41 (12)	C20—C21—H21B	109.5
C5—C11—C12	118.23 (12)	C20—C21—H21C	109.5
C10—C11—C5	122.15 (12)	H21A—C21—H21B	109.5
C10—C11—C12	119.57 (12)	H21A—C21—H21C	109.5
C8—C12—C11	118.36 (13)	H21B—C21—H21C	109.5
C9—C12—C8	123.60 (13)		
			
Br1—C9—C12—C8	−1.87 (19)	C10—C15—C16—C19	−2.4 (2)
Br1—C9—C12—C11	176.22 (10)	C11—C5—C6—C7	0.1 (2)
Br1—C9—C13—C1	1.24 (19)	C11—C10—C14—C4	179.20 (12)
Br1—C9—C13—C14	−176.59 (9)	C11—C10—C14—C13	−3.33 (19)
O3—N1—C15—C10	−179.64 (11)	C11—C10—C15—N1	−75.68 (17)
O3—N1—C15—C16	0.03 (15)	C11—C10—C15—C16	104.71 (16)
N1—O3—C17—C16	−0.55 (15)	C12—C9—C13—C1	179.42 (13)
N1—O3—C17—C18	179.89 (11)	C12—C9—C13—C14	1.6 (2)
N1—C15—C16—C17	−0.35 (16)	C13—C1—C2—C3	−0.3 (2)
N1—C15—C16—C19	178.00 (13)	C13—C9—C12—C8	179.97 (13)
C1—C2—C3—C4	0.0 (2)	C13—C9—C12—C11	−1.9 (2)
C1—C13—C14—C4	0.65 (19)	C14—C10—C11—C5	−179.58 (12)
C1—C13—C14—C10	−176.89 (12)	C14—C10—C11—C12	2.97 (19)
C2—C1—C13—C9	−177.91 (14)	C14—C10—C15—N1	110.56 (15)
C2—C1—C13—C14	−0.1 (2)	C14—C10—C15—C16	−69.05 (18)
C2—C3—C4—C14	0.6 (2)	C15—C10—C11—C5	6.85 (19)
C3—C4—C14—C10	176.60 (13)	C15—C10—C11—C12	−170.60 (12)
C3—C4—C14—C13	−0.9 (2)	C15—C10—C14—C4	−7.13 (19)
C5—C6—C7—C8	0.3 (2)	C15—C10—C14—C13	170.34 (12)
C5—C11—C12—C8	0.29 (19)	C15—C16—C17—O3	0.54 (15)
C5—C11—C12—C9	−177.91 (12)	C15—C16—C17—C18	−179.99 (15)
C6—C5—C11—C10	−177.89 (13)	C15—C16—C19—O1	0.5 (2)
C6—C5—C11—C12	−0.4 (2)	C15—C16—C19—O2	−178.75 (15)
C6—C7—C8—C12	−0.4 (2)	C17—O3—N1—C15	0.32 (14)
C7—C8—C12—C9	178.20 (14)	C17—C16—C19—O1	178.55 (13)
C7—C8—C12—C11	0.1 (2)	C17—C16—C19—O2	−0.7 (2)
C9—C13—C14—C4	178.60 (12)	C19—O1—C20—C21	−169.06 (12)
C9—C13—C14—C10	1.06 (19)	C19—C16—C17—O3	−177.91 (12)
C10—C11—C12—C8	177.84 (12)	C19—C16—C17—C18	1.6 (2)
C10—C11—C12—C9	−0.36 (19)	C20—O1—C19—O2	1.5 (2)
C10—C15—C16—C17	179.29 (13)	C20—O1—C19—C16	−177.73 (11)

### Hydrogen-bond geometry (Å, º)

**Table:**

*D*—H···*A*	*D*—H	H···*A*	*D*···*A*	*D*—H···*A*
C2—H2···N1^i^	0.95	2.54	3.4305 (19)	155
C4—H4···O2^ii^	0.95	2.58	3.1990 (18)	123

Symmetry codes: (i) *x*, −*y*+1/2, *z*−1/2; (ii) −*x*, −*y*+1, −*z*.
